# Multidrug Resistant Uropathogenic *Escherichia coli* ST405 With a Novel, Composite IS*26* Transposon in a Unique Chromosomal Location

**DOI:** 10.3389/fmicb.2018.03212

**Published:** 2019-01-08

**Authors:** Piklu Roy Chowdhury, Jessica McKinnon, Michael Liu, Steven P. Djordjevic

**Affiliations:** ^1^The ithree Institute, University of Technology Sydney, Ultimo, NSW, Australia; ^2^NSW Department of Primary Industries, Elizabeth Macarthur Agricultural Institute, Macarthur, NSW, Australia

**Keywords:** IS*26*, compound transposon, class 1 integrons, antibiotic resistance, *Escherichia coli* ST405

## Abstract

*Escherichia coli* ST405 is an emerging urosepsis pathogen, noted for carriage of *bla*_CTX-M_, *bla*_NDM_, and a repertoire of virulence genes comparable with O25b:H4-ST131. Extraintestinal and multidrug resistant *E. coli* ST405 are poorly studied in Australia. Here we determined the genome sequence of a uropathogenic, multiple drug resistant *E. coli* ST405 (strain 2009-27) from the mid-stream urine of a hospital patient in Sydney, Australia, using a combination of Illumina and SMRT sequencing. The genome of strain 2009-27 assembled into two unitigs; a chromosome comprising 5,287,472 bp and an IncB/O plasmid, pSDJ2009-27, of 89,176 bp. *In silico* and phenotypic analyses showed that strain 2009-27 is a serotype O102:H6, phylogroup D ST405 resistant to ampicillin, azithromycin, kanamycin, streptomycin, trimethoprim, and sulphafurazole. The genes encoding resistance to these antibiotics reside within a novel, mobile IS*26*-flanked transposon, identified here as Tn*6242*, in the chromosomal gene *yjdA*. Tn*6242* comprises four modules that each carries resistance genes flanked by IS*26*, including a class 1 integron with *dfrA17* and *aadA5* gene cassettes, a variant of Tn*6029*, and *mphA*. We exploited unique genetic signatures located within Tn*6242* to identify strains of ST405 from Danish patients that also carry the transposon in the same chromosomal location. The acquisition of Tn*6242* into *yjdA* in ST405 is significant because it (i) is vertically inheritable; (ii) represents a reservoir of resistance genes that can transpose onto resident/circulating plasmids; and (iii) is a site for the capture of further IS*26*-associated resistance gene cargo.

## Introduction

*Escherichia coli* are commensals of vertebrate species but pathovars have evolved that cause both intestinal (intestinal pathogenic *E. coli*; IPEC) and extraintestinal infections in humans and in food producing and companion animals. Extraintestinal pathogenic *Escherichia coli* (ExPEC) have a fecal origin but have acquired the ability to colonize urinary tract epithelium causingcystitis, pyelonephritis and sepsis and are a leading cause of meningitis and wound infections (Johnson et al., 2003; Smith et al., 2007). ExPEC are among the most frequently isolated Gram negative pathogens (Poolman and Wacker, 2016) and are increasingly resistant to broader classes of antimicrobials.

IS*26* plays a key role in the capture and spread of genes encoding resistance to different classes of antimicrobials, including drugs of last resort (Harmer et al., 2014; Chen et al., 2015; Matsumura et al., 2015; Riccobono et al., 2015; Roy Chowdhury et al., 2015; Zhang et al., 2015; Zong et al., 2015; Zurfluh et al., 2015; Agyekum et al., 2016; Harmer and Hall, 2016; Beyrouthy et al., 2017; Liu et al., 2017). It can form independently mobile compound transposons containing tandem arrays of antimicrobial resistance genes (Cain and Hall, 2012a; Venturini et al., 2013; Reid et al., 2015; Roy Chowdhury et al., 2015), often in association with class 1 integrons and diverse transposons, creating complex resistance gene loci (CRL). IS*26* can also delete and invert DNA, often modifying the conserved regions (5′- CS and 3′-CS) of class 1 integrons that are a target of polymerase chain reaction (PCR) assays used to detect them and the resistance gene cargo they carry (Dawes et al., 2010; Roy Chowdhury et al., 2011, 2015; Venturini et al., 2013; Reid et al., 2017). These events often create novel signatures that can be exploited to track the CRL they reside in (Dawes et al., 2010; Roy Chowdhury et al., 2015). Notably, IS*26* promotes: (i) the formation of cointegrate plasmids from separate replicons that carry virulence and resistance genes (Mangat et al., 2017); (ii) plasmid fitness by deleting regions of DNA that incur a fitness cost to the host (Porse et al., 2016); and (iii) the co-assembly and mobilization of resistance genes efficiently in a *recA^-^* background by preferentially integrating in regions of genomes with a pre-existing copy of IS*26* (Harmer and Hall, 2016).

Extended-spectrum beta-lactamase (ESBL)-producing, multiple drug resistant ST405-D is an emerging ExPEC pathogen (Matsumura et al., 2012; Brolund et al., 2014; Gurnee et al., 2015). A phylogenetic study of the ST405 clonal lineage, and an analysis of mobile genetic elements that play an important role in the capture and spread of antimicrobial resistance in this lineage, has not been undertaken. It is not known if ST405 is a globally dispersed emerging clone or if disease is caused by sporadic episodes by genetically divergent ST405 strains.

As part of a larger study of multiple drug resistant uropathogenic *E. coli* (UPEC) from Sydney hospitals, two strains were shown to carry a unique genetic signature in a class 1 integron created by the insertion of an IS*26* in the 3′-CS. We characterized their genome sequences using both short- and long-read genome sequencing technologies and determined both to be ST405, phylogroup D with serotype O102:H6 (ST405-D-O102:H6). These events create opportunities for the further accumulation of antimicrobial resistance genes and are being targeted for sequence analysis to better understand how multiple drug resistance is emerging in Australia. Our analyses show that the strains carried a novel IS*26*-flanked composite transposon encoding resistance to multiple antibiotics in a unique chromosomal locus. Two ST405 genomes from Denmark with near identical structures were identified by interrogating *E. coli* genomes in the RefSeq database for molecular signatures unique to Tn*6242*.

## Materials and Methods

### Strains, Isolation, and Culture Conditions

The *Escherichia coli* strains 2009-27 and 2009-30 were recovered one day apart from mid-stream urine of a patient being treated at the Sydney Adventist Hospital (SAN) in 2009. VITEK-2 species identification and phenotypic resistance profiling of the strains was performed at the SAN, testing for susceptibility to ampicillin, cephalexin, ciprofloxacin, gentamicin, nitrofurantoin, and trimethoprim using established microbiological protocols. At UTS, the strains were additionally tested using the calibrated dichotomous sensitivity (CDS) method (Bell et al., 2016) against 15 antibiotics (ampicillin 25 μg, azithromycin 15 μg, cefotaxime 5 μg, cefoxitin 30 μg, chloramphenicol 30 μg, ciprofloxacin 5 μg, co-trimoxazole 25 μg, gentamicin 10 μg, imipenem 10 μg, nalidixic acid 30 μg, nitrofurantoin 200 μg, streptomycin 25 μg, sulphafurazole 300 μg, tetracycline 10 μg, and trimethoprim 5 μg) and using *Escherichia coli* ACM 5185 as a reference control.

### DNA Purification

For PCR and Illumina sequencing, genomic DNA was extracted from 2 ml overnight cultures growing in Lysogeny Broth (LB) broth supplemented with 50 μg/ml ampicillin using an ISOLATE-II Genomic DNA kit (Bioline, Australia). Fosmid DNA and extrachromosomal DNA (IS*26* mediated DNA-loops) were extracted using the ISOLATE-II plasmid mini kit from 1.5 ml of LB culture supplemented with appropriate antibiotics. For Single Molecule Real Time (SMRT) sequencing genomic DNA from 1.8 ml of an overnight culture was isolated using a moBio genomic DNA kit (Qiagen, Germany).

### Fosmid Library Construction, Screening and PCR Conditions

A fosmid library of strain 2009-27 was constructed using the CopyControl Fosmid Library Production Kit (Epicenter) with pCC2-Fos as the vector, following the kit protocol. The library was propagated in *recA-* EPI300 chemically competent *E. coli* cells supplied by Epicenter. Five hundred individual colonies were screened for the presence of *intI1* gene using (HS915 and HS916) primers listed in Supplementary Table S1. The single fosmid clone used in the inverse PCR/loop out assay was selected on the basis of end sequences of the *intI1* positive fosmid clones and targeted PCR cartography experiments using primers listed in Supplementary Table S1.

Polymerase chain reaction consisted of 10 μl of MyTaq red (Bioline) Master Mix, 0.5 μM of each primer and 0.2 μM of template DNA in DNAse-RNAse free water (final volume 20 μl). Cycling conditions comprised of: initial denaturation step at 94°C for 2 min 30 s, 30 cycles of denaturation (94°C for 30 s) annealing (60°C for 30 s) and extension (72°C for 5 min), with a final extension cycle of 72°C for 10 min. Annealing temperatures and extension step were varied depending on the melting temperatures of the primers and expected amplicon sizes. Supplementary Table S1 lists the primers used. Supplementary Figure S1 depicts the inverse PCR strategy used to identify circular elements comprising IS*26*-associated regions of the resistance locus.

### Amplicon and Whole Genome Sequencing

Amplicons of interest were purified using a Promega Wizard^®^ SV PCR and Gel Clean Up kit following the manufacturer’s recommendations and sequenced using Sanger technology at the Australian Genome Research Facility, University of Queensland, in Brisbane. The genomes of strains 2009-27 and 2009-30 were initially sequenced at the ithree institute at UTS using a bench top Illumina MiSeq^®^ sequencer and MiSeq V3 chemistry. The sequencing library was prepared following published protocols (Darling et al., 2014b). The 400 nt long paired end reads were assembled using an A5-MiSeq (ngopt_a5pipeline_linux-x64_20130919*)*.

DNA from strain 2009-27 was also sequenced using a PacBio RSII instrument at the Ramaciotti Centre for Genomics, UNSW, Sydney. Sequencing reads were assembled with HGAP and Quiver. The plasmid sequence was closed using Circlator (Hunt et al., 2015) and polished with Illumina MiSeq raw read sequences using PILON (Walker et al., 2014). Whole genome sequences of strain 2009-30 (Illumina) and strain 2009-27 (PacBio) are deposited in GenBank under accession numbers NXEQ00000000 and NXER00000000.

Preliminary genome annotations were generated using an online version of RAST (Overbeek et al., 2014) using FigFAM release 70. Putative virulence and antimicrobial resistance genes were identified using stand-alone BLASTn analyses and an in-house database, prior to manual verification using NCBI-ORF finder. Legitimate ORFs showed > 95% sequence similarity (E-value 0.001) across 100% of the query sequence. Sequences of interest were characterized using iterative BLASTn and BLASTp searches (Altschul et al., 1997).

### Bioinformatics

Phylosift (Darling et al., 2014a) was used to infer phylogenetic relationships among ST405 strains 2009-27 and 2009-30 and 328 assembled *E. coli* ST405 genomes in Enterobase^1^ as of June 29, 2017. A subset of 24 ST405 genomes that clustered together using marker gene phylogeny based PhyloSift analysis were subjected to core-genome alignment based phylogeny analysis using Parsnp (Treangen et al., 2014)^2^ and selecting the -c (all genomes) and -x recombination filter flags. The assembled genome of biosample SAMEA4559509 (sample ERS1458688) was downloaded from Enterobase and used as a reference. Comparative, whole genome analyses were performed using Mauve (Darling et al., 2010). A SNP based phylogenetic tree of plasmid backbone sequences extracted from the longest locally collinear block derived from a progressiveMauve alignment was constructed using algorithms in FastTree_accu^3^ and visualized using FigTree version 1.4.0.^4^

Multilocus sequence typing was performed *in silico* (eMLST) using the PubMLST database^5^ and the Achtman *E. coli* MLST scheme^6^ (Jolley and Maiden, 2010). Average nucleotide identity (ANI) was calculated using the ANI calculator portal at.^7^ SMRT sequences from strain 2009-27 were used or all comparative genomic analyses.

## Results

### Genomic Analysis of Strains 2009-27 and 2009-30

Illumina sequences of strains 2009-27 and 2009-30 assembled into 243 scaffolds (N50 = 89298; 47 × median coverage) and 191 scaffolds (N50 = 91427; 45 × median coverage), respectively. SMRT sequences of strain 2009-27 (N50 = 20303) assembled into two unitigs; a chromosome comprising 5,287,472bp and a plasmid (pSDJ2009-27) of 89,176 bp. *In silico* analyses showed that strains 2009-27 and 2009-30 were ST405, serotype O102:H6 and phylogroup D (ST405-D-O102:H6). Both strains were phenotypically resistant to ampicillin, azithromycin, streptomycin, trimethoprim and sulphafurazole. ANI analysis of the two genomes indicated that strains 2009-27 and 2009-30 are indistinguishable from one another (99.9% mean identity and 100% median identity).

### Plasmid Analysis

pSDJ2009-27 typed as IncB/O/K/Z in plasmidFinder. The *repA* gene, encoding replication initiator protein, in pSDJ2009-27 was identical to *repA* in pR3521 (GU256641), a self-transmissible multi-drug resistant IncB/O plasmid encoding *bla*_ACC-4_, *bla*_SCO-1_, and *bla*_TEM-1_ from a patient in Greece (Papagiannitsis et al., 2011). No antimicrobial resistance genes were found on pSDJ2009-27 but it contained IncI1 plasmid-related *tra-*genes essential for conjugative transfer. Notably, the sequence of *repA* was also identical to *repA* in pO26-Vir (FJ386569.1) and showed ≥ 99% similarity to *repA* sequences in plasmids pR3521 (GU256641), pEC3I (KU932021), pHUSEC411-like (HG428756), and pHUSEC41-1 (HE603110) associated with IPEC. BLAST analysis showed that pSDJ2009-27 shared ≥ 99% identity over 89% of the query sequence with pO26-Vir (FJ386569.1) from Shiga-toxin producing O26:H11 strain H30 (Fratamico et al., 2011) (Supplementary Figure S2). A progressiveMauve alignment (Supplementary Figure S3) of the plasmid sequences with near identical *repA* genes revealed a ∼60 kb conserved fragment of high sequence similarity. Phylogenetic analysis of the aligned regions of these plasmids (Supplementary Figure S4) confirmed that pO26-Vir was the most closely related plasmid and originated from a recent common ancestor. A comparative bi-directional peptide BLAST analysis of all ORFs identified by RAST within the 60 kb fragment in pSDJ2009-27 with those on pO26-Vir, pEC3I, pHUSEC411-like, and pHUSEC41-1 indicated that they contain genes for IncI plasmid maintenance, stability and transfer (Supplementary Table S2). pO26-Vir is a large (168-kb) mosaic plasmid that carries putative EHEC virulence genes *toxB, katP, ehxA*, and *espP.* Notably, ∼45-kb of pO26-Vir containing these genes is absent in pSDJ2009-27 (Supplementary Figure S4 and Supplementary Table S3). We have recently described pO26-CRL (Venturini et al., 2010) (represented as the inner green circle in Supplementary Figure S2) from an O26:H- EHEC isolated from a patient with hemorrhagic colitis and pO26-CRL-125 (Venturini et al., 2013), that were also similar to pO26-Vir (Supplementary Figure S2).

### Characterisation of the CRL in Strain 2009-27

The class 1 integron in strains 2009–27 and 2009–30 is flanked by direct copies of IS*26*, one of which was located 209 nucleotides downstream from the start site of the *sul1* gene in the 3′-CS, rendering *sul1* inactive. The variable region of the integron carried *dfrA17* and *aadA5* genes encoding resistance to trimethoprim and streptomycin. A PCR developed previously to identify novel IS*26*-mediated genetic signatures within the 3′-CS of class 1 integrons using a primer in *attI1* (L1 primer) in the 5′-CS and a primer in IS*26 tnpA* (JL-D2) (Dawes et al., 2010; Roy Chowdhury et al., 2015) generated a unique amplicon of 2425-bp and this was exploited to track this unique genetic locus.

**FIGURE 1:**
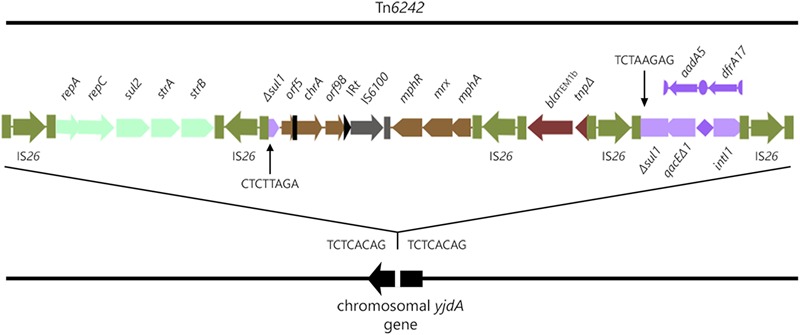
Structure of Tn*6242* in the chromosome of ST405 strain 2009-27.

Analysis of the SMRT sequences from strain 2009–27 indicated it had a chromosomally located CRL, 19,774-bp [nucleotides 1732675 to 1752448, unitig_0 (NXER00000000)] in length. The CRL is flanked by direct copies of IS*26* and located within *yjdA* in the chromosome, a site not known for insertion of laterally acquired DNA (Figure 1). An eight nucleotide direct duplication (TCTCACAG) was identified (Figure 1) flanking the CRL indicating that IS*26* was responsible for its movement. The IS*26*-flanked transposon is registered as Tn*6242* in the transposon database.^8^

The structure of the CRL is modular, comprising five copies of IS*26* that flank modules 1–4 containing combinations of different antimicrobial resistance genes. Module 1 comprises *repA-repC-sul2-strA-strB* genes bounded by two inwardly orientated IS*26* elements, a structure that forms part of the Tn*6029* family of transposons (Dawes et al., 2010; Yau et al., 2010; Cain and Hall, 2012b; Roy Chowdhury et al., 2015). Module 2 is 6924-bp and comprises Δ*sul1*, Δ*orf5* interrupted by the inverted repeat of Tn*501* (IR_Tn_*_501_*), *chrA, orf98*, the IR*t* inverted repeat typical of clinical class 1 integrons, IS*6100*, and the macrolide resistance operon *mphR-mrx-mphA*. The sequence between *Δsul1* and the macrolide resistance operon is frequently reported in association with class 1 integrons on different *E. coli* plasmids from geographically distant sources (GenBank accession numbers LT632321.1; FQ482074.1; EU935739.1). Beyond the macrolide resistance operon is a third 1402-bp Tn*2*-derived IS*26* flanked module (module 3) comprising *bla*_TEM-1b_ and a ΔTn*2* transposase gene. *bla*_TEM-1b_-associated Tn*2*-derivatives typically form part of the Tn*6029* family of transposons (Roy Chowdhury et al., 2015), although this 1402-bp fragment is unique (see later). A class 1 integron with a truncated copy of *intI1* comprises module 4 in the CRL (Figure 1). *ΔintI1* comprises 657 nucleotides and the deletion event presumably was associated with insertion of IS*26*. The recombination functional of *ΔintI1* is non-functional in strains 2009-27 and 2009-30 and the integron is not expected to integrate or exchange gene cassettes. The P_C_ promoter remains intact, however, and is expected to induce the expression of *aadA5* and *dfrA17* resistance genes, an observation consistent with phenotypic resistance to trimethoprim and streptomycin.

### Prevalence of Tn*6242*

A 24.1-kb DNA fragment comprising 19.8-kb of the CRL and about 5 kb flanking both ends of *yjdA* gene was used to interrogate the GenBank RefSeq database. Two *E. coli* genomes (accession numbers NZ_KE701406.1 and NZ_KE699379.1) showed ≥ 99% sequence identity across 100% of the input query sequence (Supplementary Figure S5). NZ_KE701406.1 and NZ_KE699379.1 were ST405-D-O102:H6 and were from urine and blood of unrelated Danish patients, respectively. Each strain carried the four resistance gene modules of Tn*6242*, including the class 1 integron and associated cassette array, on one supercontig. Barring small deletions that may be products of assembly errors, these sequences were indistinguishable (Supplementary Figure S5).

### Comparative Phylogenomics of ST405 Strains

**FIGURE 2:**
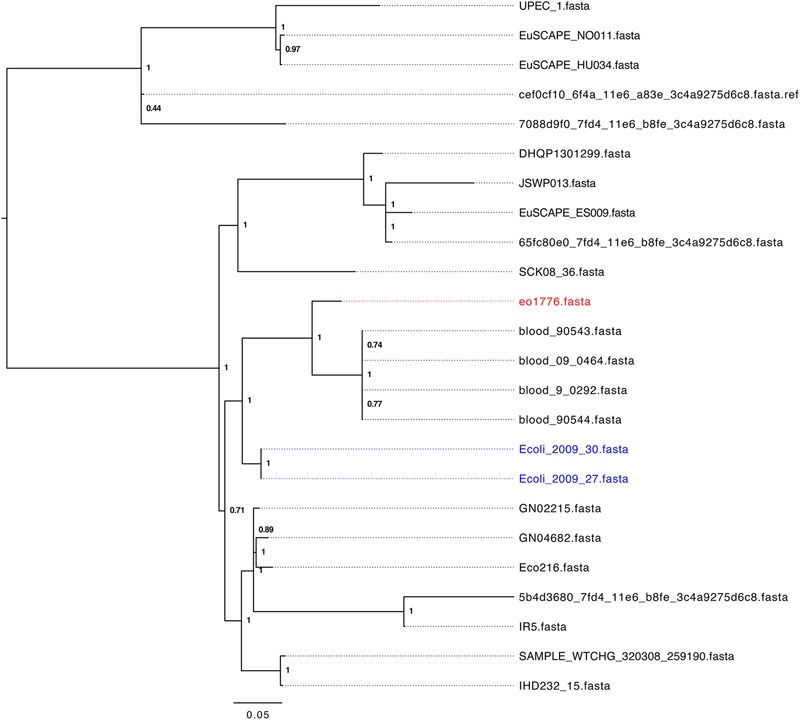
Parsnp tree analysis of E. coli ST405 genomes phylogenetically related to strain 2009-27.

To identify a cohort of ST405 strains related to strains 2009-27 and 2009-30 we initially performed a marker gene based phylogeny analysis of 328 assembled *E. coli*-ST405 genomes from Enterobase (26th July, 2017) using Phylosift (Darling et al., 2014a). Strains 2009-27 and 2009-30 clustered with 24 ST405 strains (Supplementary Figure S6) from human infections from unrelated geographic locations. Core genome alignment based phylogeny analysis of these 24 ST405 strains (Figure 2) identified e01776 to be the closest relative of 2009-27 (1414 SNP differences) while four strains, from patients in the United States with blood infections, showed between 1662 and 1665 SNP differences. Fifteen of the 24 strains (Supplementary Table S4) had an identical class 1 integron and the remaining nine had *intI1* interrupted by IS*26.* The insertion site of IS*26* in *intI1* occurred at two separate locations both of which were different to Δ*intI1* in Tn*6242* (Supplementary Table S4).

A BLASTn search (≥ 95% identity over 100% of the query sequence) of antimicrobial resistance and virulence genes in the genome of 2009-27 against an in-house database of resistance genes identified *bla*_CTX-M88_ and *bla*_OXA-1_ in the five ST405 genomes that clustered with 2009-27 (Table 1). Several of the five *E. coli* ST405 genomes carry all the resistance genes characteristic of Tn*6242* (*dfrA17, aadA5, sul1, mph(A), sul2, strA*, and *strB*) on the same scaffold. Supplementary Table S5 depicts an alignment of the 19.7-kb region spanning Tn*6242* with the seven closely related *E. coli* ST405 genomes identified here. The four blood-borne *E. coli* ST405 strains had a copy of the class 1 integron with a Δ*intI1* variant, as well as the IS*6100*-associated macrolide resistance module that abuts the 3′-CS with *orf5, chrA*, and *orf98* genes on the same scaffold. Strain eo1776 did not carry the macrolide resistance module. Notably, all five strains contained an intact copy of *yjdA* and had a variable repertoire of virulence genes (Supplementary Table S6). Comparative plasmid profiling of the five closely related genomes with strain 2009-27 suggested that *E. coli* ST405 strains 09-90543 and 09-90544 also had an IncB/O/K/Z plasmid replicon in addition to IncFII and pCol replicons, while blood isolates 09-0464 and blood 09-0292 carried IncFII and pCol replicons. Strain eo1776 was unusual in that it only carried an IncFII replicon. These data suggest that the class 1 integron may be associated with IncFII plasmids in this collection.

**Table 1:** Resistance genes in ST405 genomes.

Resistance genes	2009-27	blood-09-0464	blood-9-0292	blood-90543	blood-90544	eo1776
*tet(B)*(AF326777)	x	100	100	100	100	100
*tet(A)*(AJ517790)	x	100	100	100	100	x
*sul2* (GQ421466)^∗^	99.63	100	100	100	100	x
*sul2* (HQ840942)^∗^	100	99.63	99.63	99.63	99.63	x
*strA* (AF321551)^∗^	100	100	100	100	100	100
*strB* (M963920)^∗^	99.88	99.88	99.88	x	x	x
*bla*_TEM-1A_^∗^ (HM749966)	99.65	x	x	99.77	99.65	x
*bla*_OXA-1_ (J02967)	x	100	100	100	100	100
*bla*_CTX-M-88_ (FJ873739)	x	99.89	99.89	99.89	99.89	99.89
*aac*(6′)*Ib-cr* (DQ303918)	x	100	100	100	100	100
*aacA4* (KM278199)	x	99.64	99.64	99.64	99.64	99.64
*dfrA17* (FJ460238)^∗^	100	100	100	100	100	100
*aadA5* (AF137361)^∗^	100	100	100	100	100	100
*sul1* (CP002151)^∗^	100Δ	100	100	100	100	100
*mph(A)* (D16251)^∗^	100	100	100	100	100	x

*Numbers indicate the percentage identity across 100% of the input query sequence. An asterisk (^∗^) indicates the resistance genes present within the boundaries of Tn*6242*. Δ indicates partial deletion at one end of the gene due to breaks in the scaffolds of the genome sequence.*

### Evolution of Tn*6242*

**FIGURE 3:**
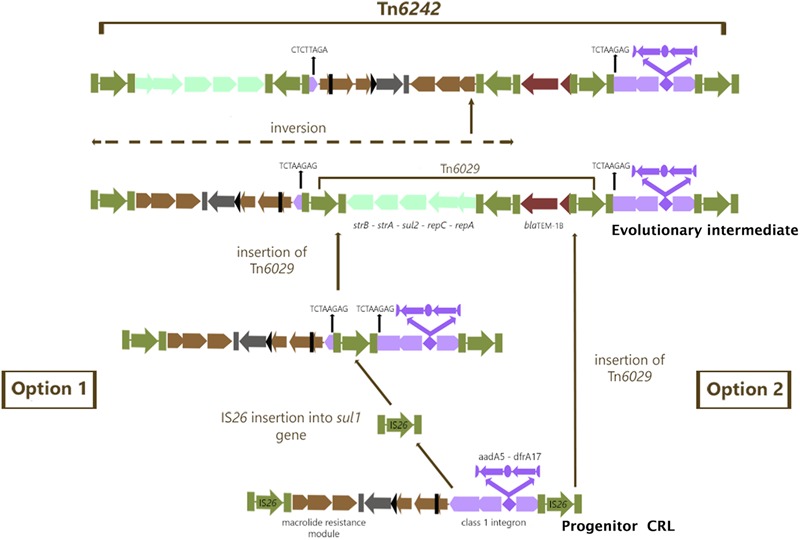
Genealogy of Tn*6242.* Green color arrows bounded by green bars indicate IS*26*. The aqua colored group of arrows indicate module 1 consisting of the *repA-repc-sul2-strA-strB* genes, the light brown colored arrows indicate module 2 containing *orf5-chrA*-IS*6100*-*mphR-mrx-mphA* genes, the chocolate colored gene indicates module 3 with the *bla*_TEM1b_ gene and the violet colored arrows indicate the class 1 integron associated Δ*intI1-dfrA17-aadA5-qacE*Δ*1-sul1*Δ genes described as module 4. For more details of the genes and their orders please refer to Figure 1.

In module 4 of Tn*6242* the sulfonamide resistance gene *sul1* is interrupted by a fragment of DNA encoding *bla*_TEM-1B_ and the macrolide resistance operon in module 1 is flanked by oppositely facing copies of IS*26*. The reverse complement of the last eight bases of the *sul1* gene (TCTAAGAG) flanks one arm of module 2 while the complimentary sequence CTCTTAGA flanks the other end (Figure 1) of module 3. These observations, coupled with the fact that the orientation of module 2 and 3 is inverted in relation what is often seen in typical clinical class 1 integrons, suggest that modules 2 and 3 have undergone an IS*26*-mediated inversion, an event that can occur in regions of DNA flanked by oppositely facing copies of IS*26* (Roy Chowdhury et al., 2015). To explain the genealogy of Tn*6242* (Figure 3), a hypothetical progenitor of 10,698 bp, comprising an inverted copy of modules 2 and 3 that abuts the 3′-CS of the class 1 integron with *sul1* intact, was assembled *in-silico*. We also removed a copy of IS*26* that presumably inserted into the 3′-end of *sul1* in the progenitor. A BLASTn analysis was undertaken to determine if our hypothetical progenitor has been described previously. The analysis identified matches (100%) to integrons in plasmids ‘p’ from strain NCTC 13441 (LT632321.1), pETN48 from *E. coli* ST405-O102 (FQ482074.1), pEK499 (EU935739.1) from ST131-O25:H4, and pEC958 from *E. coli* ST131-O25b:H4 (HG941719.1) from Australia. From the progenitor sequence there are several features of Tn*6242* that indicate it may have formed in one of two ways. Option 1 in Figure 3 depicts a copy of IS*26* inserting into *sul1*, generating a target site 8-bp duplication (TCTAAGAG), followed by the insertion of a Tn*6029*-family transposon (Harmer and Hall, 2015, 2016). In option 2 (Figure 3), a Tn*6029*-family transposon, rather than IS*26*, inserted in the *sul1* generating the same 8-bp target site duplication. The resulting structure, identified as the evolutionary intermediate in Figure 3, positions 209 nucleotides at the 3′-end of *sul1* gene away from the rest of the *sul1* gene. Tn*6029*- and Tn*6026*-family transposons are widely dispersed within *Salmonella enterica* and *E. coli* populations in Australia and are known to be associated with plasmid-encoded, mercury resistance transposons (Dawes et al., 2010; Venturini et al., 2013). Strain 2009-27 does not contain a mercury resistance transposon, indicating that the formation and mobilization of the CRL was driven by IS*26*. In the evolutionary intermediate, two inwardly facing copies of IS*26* flank *repA-repC*-*sul2*-*strA-strB* and the *mphR-mrx-mphA* module beyond with the intervening sequence (option 2, Figure 3), enabling us to trace an IS*26*-mediated inversion event, generating the structure seen in Tn*6242*.

### Generation of Laterally Mobile Translocatable Units From Tn*6242*

It is conceivable that Tn*6242* can generate multiple translocatable units (TUs), each mobilizing a different combination of resistance gene module flanked by direct copies of IS*26*. To investigate this, we cloned the CRL into a pCC2Fos fosmid vector and propagated the fosmid in a *recA-* background (*E. coli* strain EP1300). An inverse PCR strategy (Supplementary Figure S1) was used to examine the ability of the module containing the macrolide resistance gene cluster and the integron-containing module to be rescued as a translocatable unit (TU). Sanger sequencing of inverse PCR amplicons that span across the IS*26* junctions indicated that all three TUs were generated (Supplementary Figure S7). Therefore, pSDJ2009-27 could act as a vector for Tn*6242* and generate different combinations of laterally mobile resistance gene modules flanked by direct copies of IS*26* from Tn*6242*.

## Discussion

Genome sequence analyses of ST405-D-O102:H6 strains 2009-27 and 2009-30 from the urine of the same patient that were resistant to ampicillin, azithromycin, streptomycin, trimethoprim, and sulphafurazole is presented here. In these Sydney strains, we describe the structure of a novel IS*26*-derived and laterally mobile compound transposon, identified here as Tn*6242*. Tn*6242* comprises a class 1 integron, a variant of Tn*6029*, and an *mphA* operon and these are sufficient to encode resistance to a panel of antibiotics described above. Notably, Tn*6242* is located within a unique chromosomal location in *yjdA*. We predict a series of genetic events that led to the formation of Tn*6242* and showed how the genetic signatures formed during its evolution were exploited to identify two MDR strains of ST405 from Denmark that carry minor variants of Tn*6242* in *yjdA*.

Our data suggest that this lineage of MDR ST405-D-O102:H6 may be globally disseminated. Analyses of phylogenetically related ST405 genomes in Enterobase indicate that they carry CRL with similar genetic cargo but were not associated with *yjdA*. Since most IS*26*-associated resistance regions are plasmid-encoded, the evolutionary events that created Tn*6242* in *yjdA* are likely to be recent. It will be important to monitor IS*26*-flanked CRL that associate with *yjdA* as it may represent a novel and emerging hotspot for the insertion of other IS*26-*flanked elements in the chromosome of clinically important *Enterobacteriaceae*.

The *repA-repC-sul2-strA-strB* module in Tn*6242* is flanked by two inwardly facing IS*26* elements and shares 99% sequence identity with structures found in pSRC26 (GQ150541), pSRC27-H (HQ840942.1) (Cain and Hall, 2012a), pO26-CRL (GQ259888.1), pO26-CRL-125 (KC340960.1), and pO111-CRL-115 (KC340959.1) (Venturini et al., 2013), all reported in Australia. However, the Tn*2* transposon in the *bla*_TEM-1b_ module is 1402-bp, and is much smaller than Tn*2* variants in Tn*6029* (1916-bp), Tn*6029B* (1831-bp), or Tn*6029C* (1820-bp) (Roy Chowdhury et al., 2015). BLAST analysis confirmed that the 1402-bp variant is novel suggesting that the deletion likely arose as a consequence of the inversion event described above. Notably, sequences that aligned with ≥ 99.9% identity to the input query were plasmids from Australia that carry derivatives of Tn*6029B*. Collectively, our data suggests that Tn*6242* may be a locally derived IS*26*-flanked transposon.

Finally, the backbone of a resident IncB/O/K/Z plasmid (pSDJ2009-27) showed ≥ 99% sequence identity with pO26Vir from an O26:H11 Shigatoxigenic *E. coli* and pO26-CRL from an enterohemorrhagic *E. coli* O26: H^-^ strain. STEC O26:H11/H- has been associated with Shiga toxin production and foodborne illness and are the second most frequent cause of EHEC-associated food-borne illness globally (Lajhar et al., 2017). pO26-Vir and pSDJ2009-27 carry a type IV pilus biosynthesis locus (*pil*) comprising *pilL* - *pilV* that may be important for biofilm formation (Srimanote et al., 2002). ExPEC have a fecal origin and plasmids circulating in IPEC may adapt to play important roles in ExPEC virulence. To our knowledge, such an observation has not been reported and this is the first time an IPEC virulence plasmid has been identified in uropathogenic *E. coli*.

## Ethics Statement

This study does not require any ethics approval. The isolates in the study came from a larger collection of *E. coli* isolates set aside by the hospital microbiology laboratory for our research. They were collected in accordance with Hospital policy as part of routine microbiology of hospital patients. We received de-identified isolates so patient identity is not known. There was no requirement for ethics approval on de-identified samples when they collected.

## Author Contributions

PRC and SD conceptualized and designed the study, and co-wrote the manuscript. PRC and JM analyzed the data. JM also assisted in the generation of the first draft of the manuscript. ML was involved in the generation of short and long read sequencing of the genomes.

## Conflict of Interest Statement

The authors declare that the research was conducted in the absence of any commercial or financial relationships that could be construed as a potential conflict of interest.

## Footnotes

1 http://enterobase.warwick.ac.uk/

2 https://github.com/marbl/Parsnp

3 http://darlinglab.org/blog/2015/03/23/not-so-fast-fasttree.html

4 http://tree.bio.ed.ac.uk/software/figtree/

5 http://pubmlst.org/

6 http://mlst.warwick.ac.uk/mlst/

7 http://enve-omics.ce.gatech.edu/ani/index

8 https://transposon.lstmed.ac.uk/tn-registry

## Supplementary Material

FIGURE S1 Inverse PCR strategy used to identify IS*26*-associated mobile elements.

FIGURE S2 Alignment of regions of pO26-vir, pSDJ2009-27 and pO26_CRL which share ≥ 99% sequence identity over specific regions. Plasmid pO26-vir, represented in the outermost circle has color coded open reading frames indicating position of specific gene/ gene clusters. The maroon circle indicates regions present in pSDJ2009-27 and the green innermost green circle indicate pO26_CRL.

FIGURE S3 Progressive Mauve alignments of pSDJ2009-27 and plasmids with identical *repA* genes. The ∼ 60-kb region of homology is highlighted with a red box.

FIGURE S4 SNP tree of plasmids related to pSDJ2009-27.

FIGURE S5 Alignment of Tn*6242* and flanking sequences with NZ_KE701406.1 and NZ_KE699379.1.

FIGURE S6 Phylosift analysis of ST405 strains in Enterobase. Sydney ST405 strains included in this study are highlighted in red. No geographic location data was available for 12 strains.

FIGURE S7 Structure of Tn*6242* with red arrows adobe specific modules which were experimentally proven to loops out under stress, with sanger sequencing of inverse PCR amplicons. Primers on top of the red arrows indicate the primers used in the PCR cartography experiment to select the *intI1* positive fosmid clone for the looping out experiment.

TABLE S1 Primers and PCR conditions used in this work.

TABLE S2 Comparative peptide BLAST analysis of the ORFs predicted from the sequence of pSDJ2009-27 by RAST and other closely related plasmids.

TABLE S3 RAST annotation of SDJ2009-27.

TABLE S4 Relative abundance of resistance genes in ST405.

TABLE S5 BLASTn alignment of Tn*6242* in ST405 genomes.

TABLE S6 Stand-alone BLASTn analysis of virulence genes in ST405. “1” indicates presence and “0” indicates absence of an identical copy of the gene in the respective genome.
