# Supplementary figures and images for: Multidrug Resistant Uropathogenic Escherichia coli ST405 With a Novel, Composite IS26 Transposon in a Unique Chromosomal Location
Source: Front Microbiol. 2019 Jan 8;9:3212. doi: 10.3389/fmicb.2018.03212 (PMC6331395; doi:10.3389/fmicb.2018.03212)

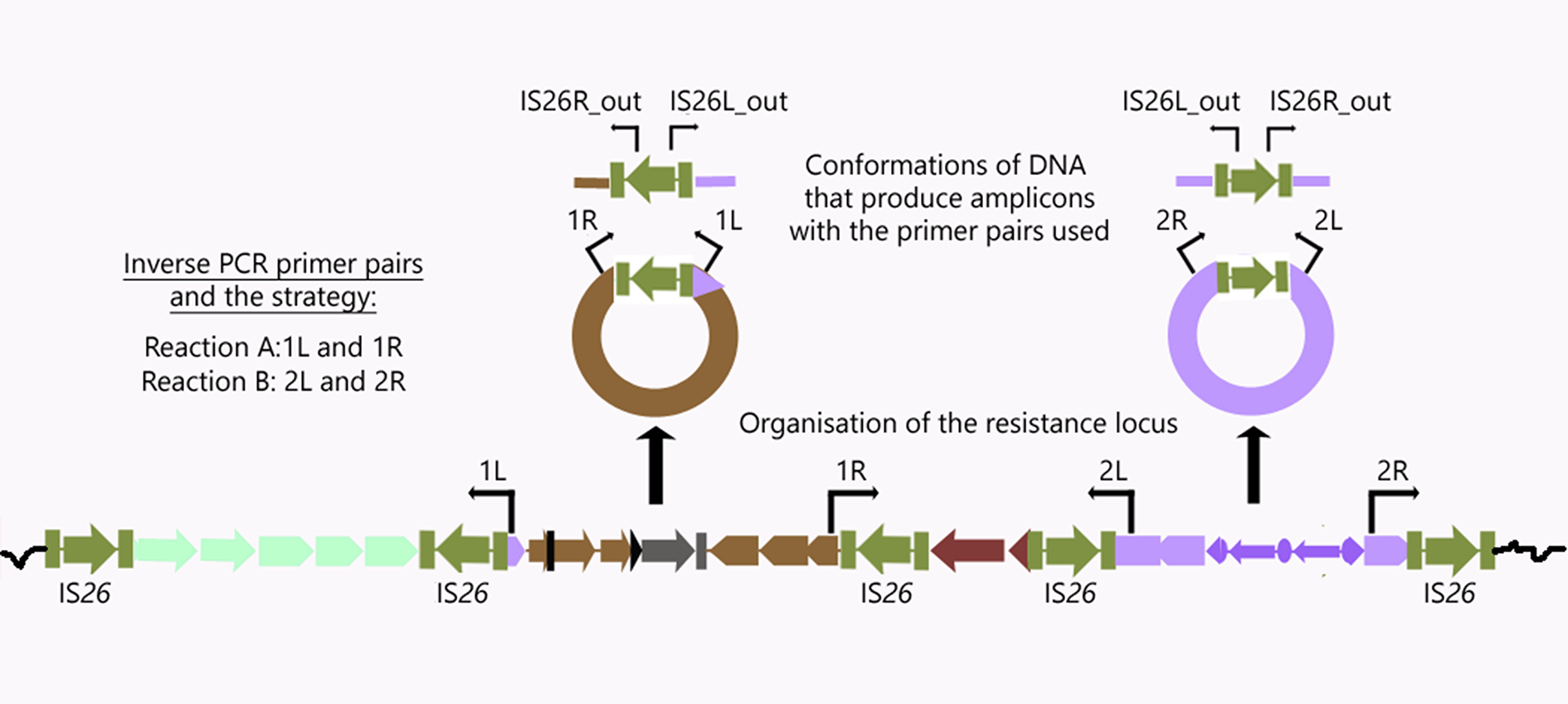

Supplement: FIGURE S1 — Inverse PCR strategy used to identify IS26-associated mobile elements. [file Image_1.TIF]

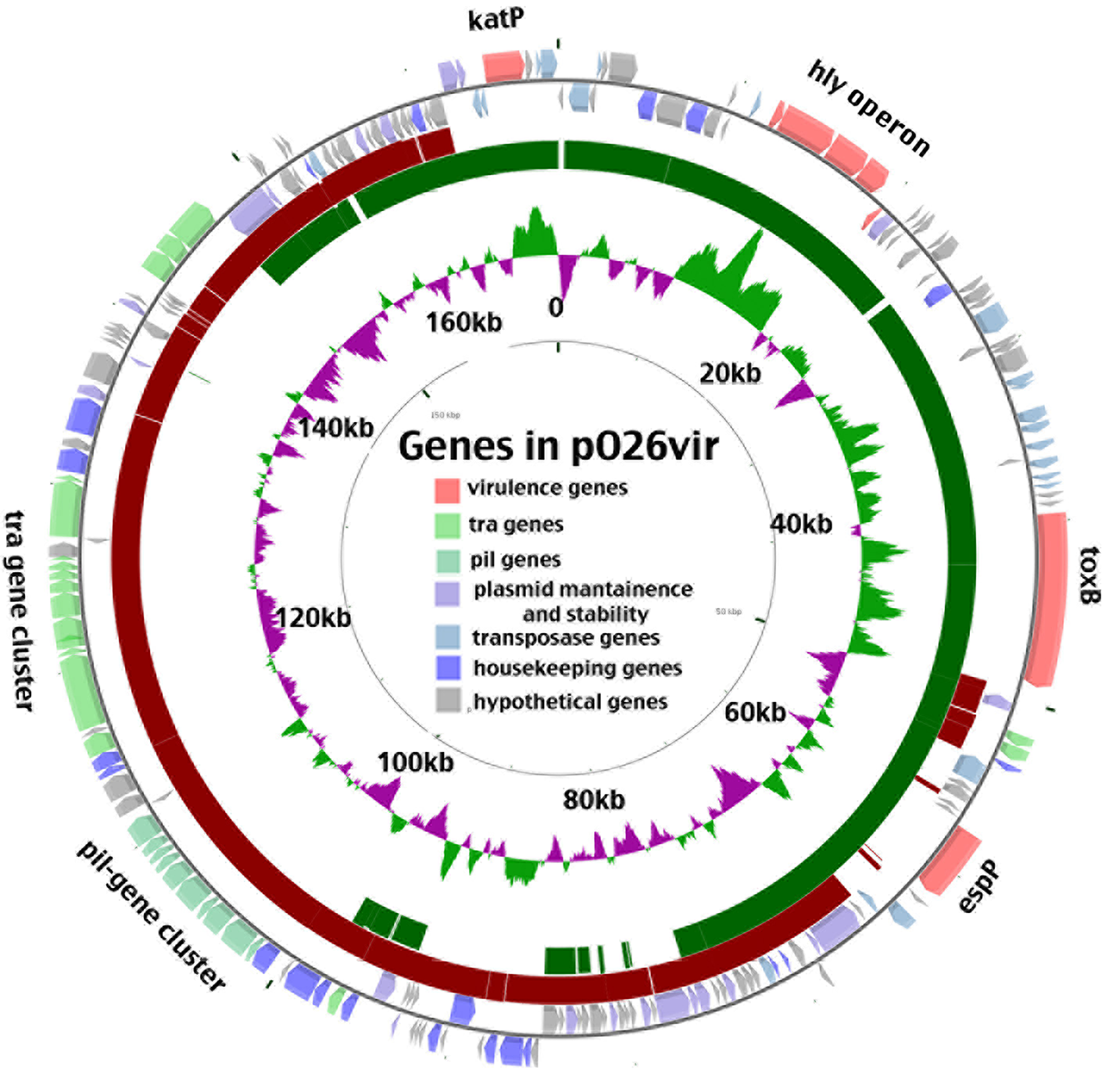

Supplement: FIGURE S2 — Alignment of regions of pO26-vir, pSDJ2009-27 and pO26_CRL which share ≥ 99% sequence identity over specific regions. Plasmid pO26-vir, represented in the outermost circle has color coded open reading frames indicating position of specific gene/ gene clusters. The maroon circle indicates regions present in pSDJ2009-27 and the green innermost green circle indicate pO26_CRL. [file Image_2.TIF]

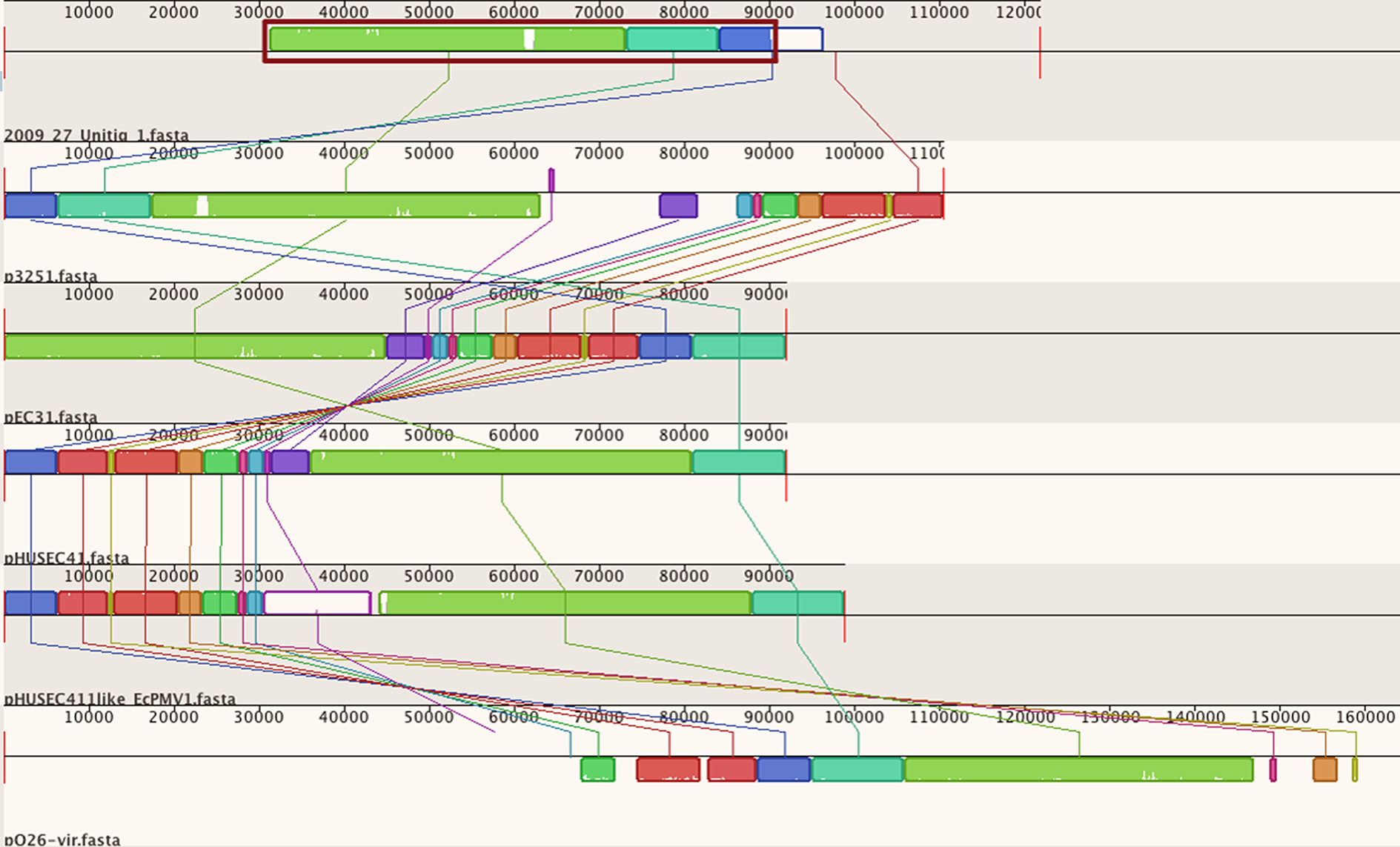

Supplement: FIGURE S3 — Progressive Mauve alignments of pSDJ2009-27 and plasmids with identical repA genes. The ∼ 60-kb region of homology is highlighted with a red box. [file Image_3.TIF]

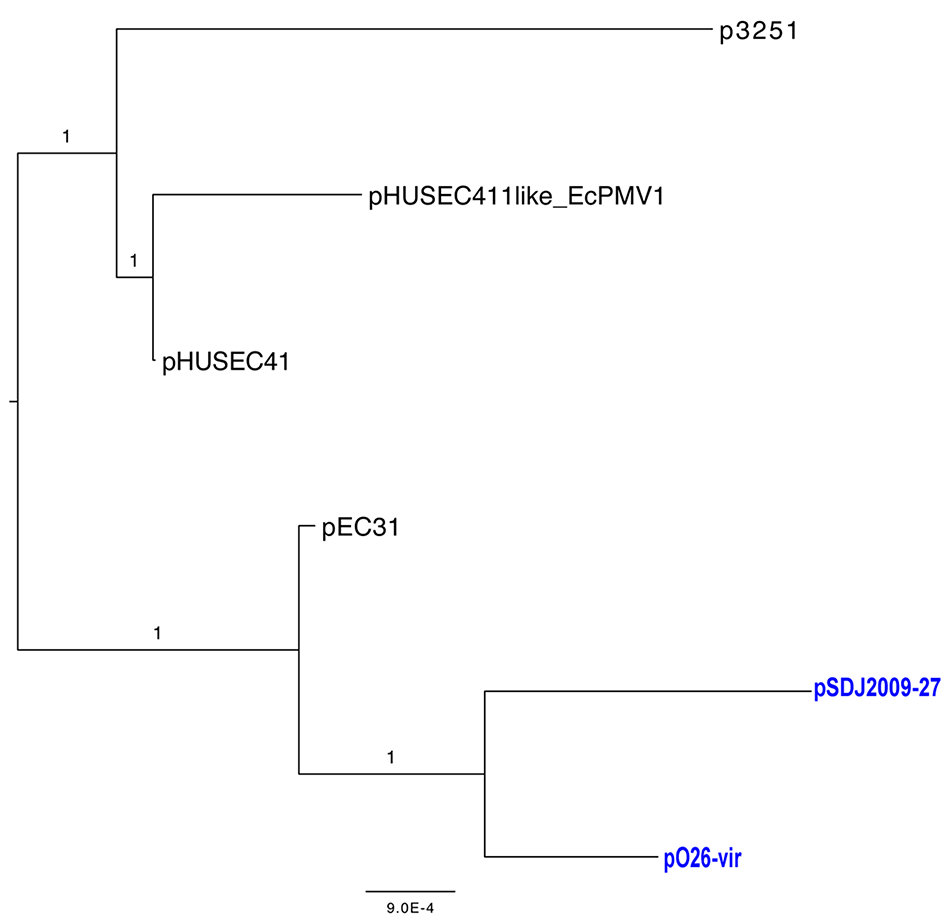

Supplement: FIGURE S4 — SNP tree of plasmids related to pSDJ2009-27. [file Image_4.TIF]

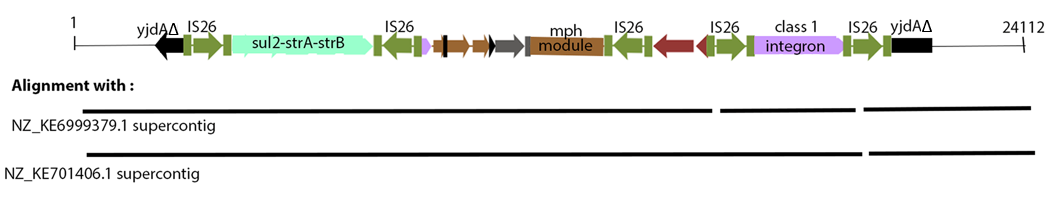

Supplement: FIGURE S5 — Alignment of Tn6242 and flanking sequences with NZ_KE701406.1 and NZ_KE699379.1. [file Image_5.tif]

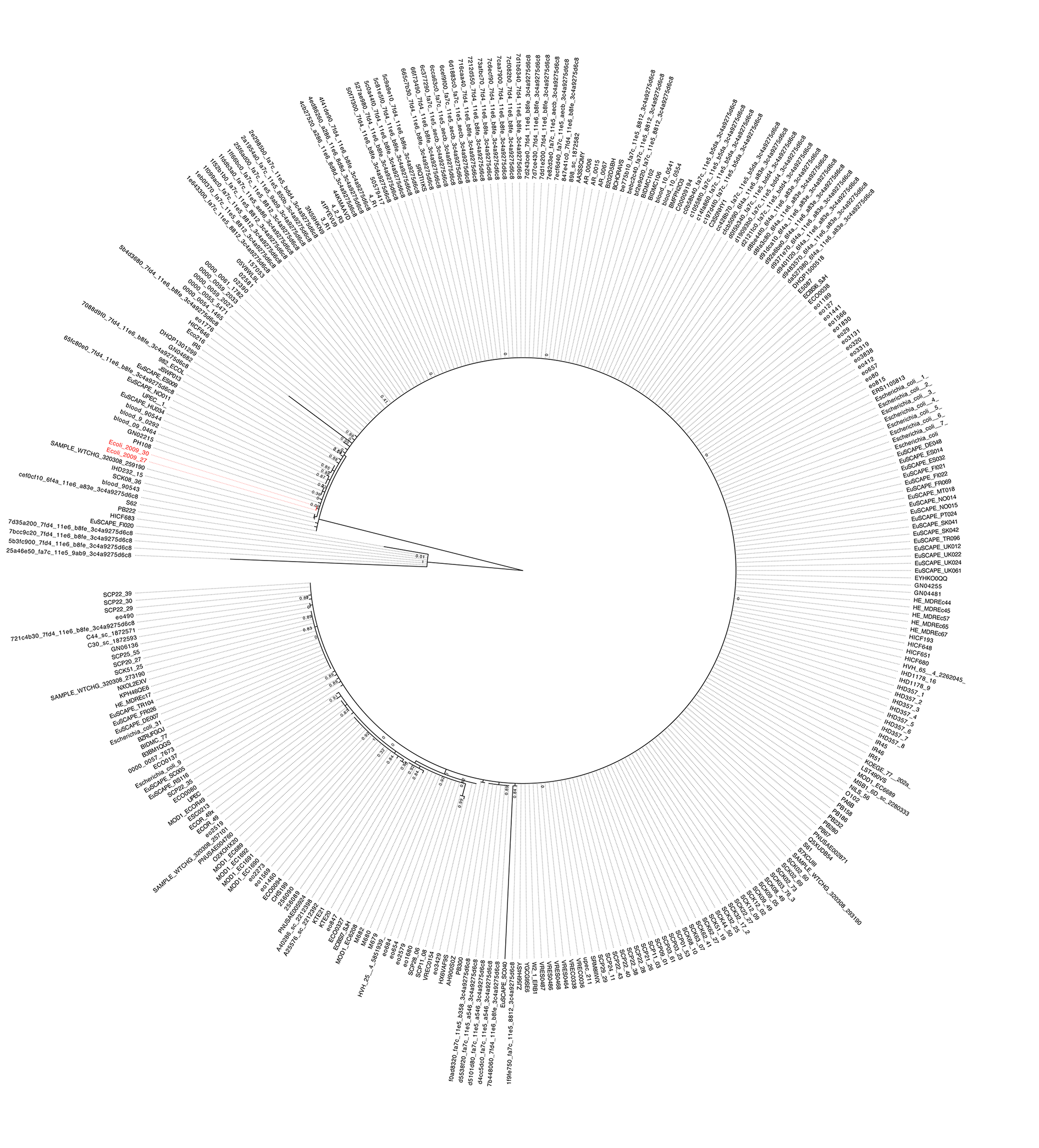

Supplement: FIGURE S6 — Phylosift analysis of ST405 strains in Enterobase. Sydney ST405 strains included in this study are highlighted in red. No geographic location data was available for 12 strains. [file Image_6.TIF]

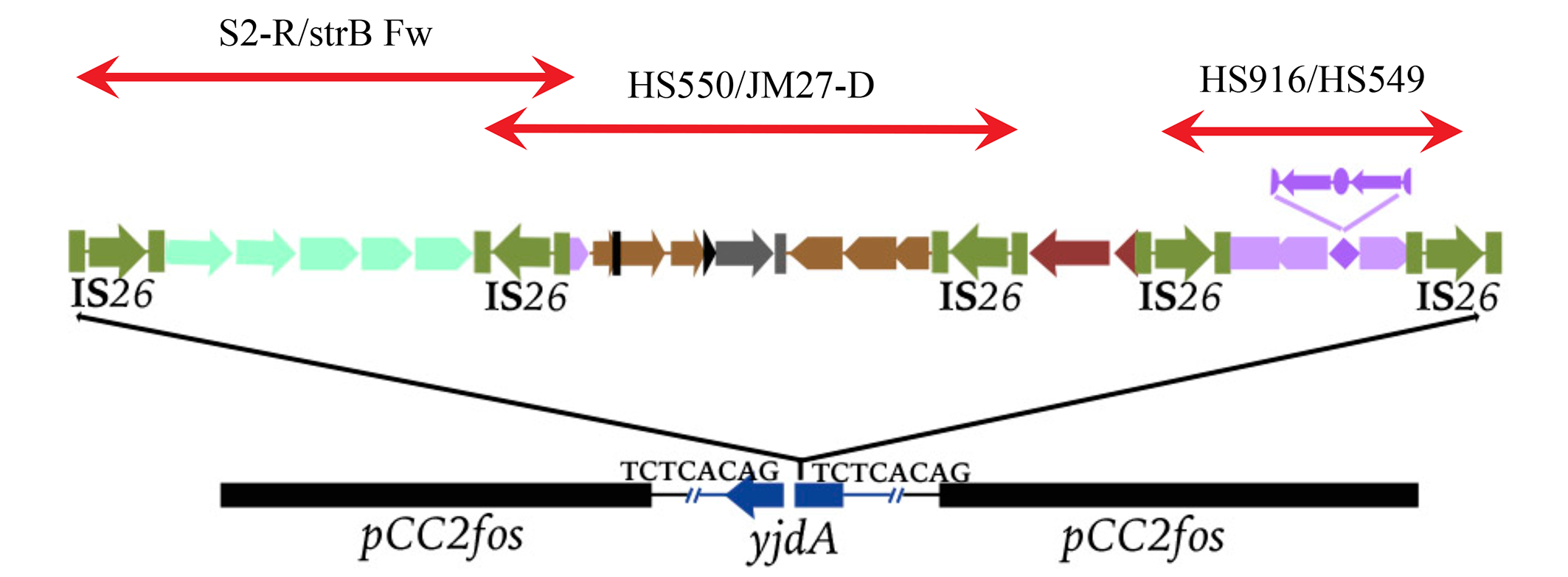

Supplement: FIGURE S7 — Structure of Tn6242 with red arrows adobe specific modules which were experimentally proven to loops out under stress, with sanger sequencing of inverse PCR amplicons. Primers on top of the red arrows indicate the primers used in the PCR cartography experiment to select the intI1 positive fosmid clone for the looping out experiment. [file Image_7.TIF]
