# Supplementary material for: Multidrug Resistant Uropathogenic Escherichia coli ST405 With a Novel, Composite IS26 Transposon in a Unique Chromosomal Location
Source: Front Microbiol. 2019 Jan 8;9:3212. doi: 10.3389/fmicb.2018.03212 (PMC6331395; doi:10.3389/fmicb.2018.03212)
Supplement: TABLE S1 — Primers and PCR conditions used in this work. [file Table_1.DOCX]

**File S1: Table.** Primers and PCR conditions used in this work

| Loop | Primers | Primer Sequence | Melting Temperature (°C) | PCR Amplicon (bp) |
| --- | --- | --- | --- | --- |
| 1 | HS549 | ACTAAGCTTGCCCCTTCCGC | 65 | 1825 |
|  | HS916 | TTCGTGCCTTCATCCGTTTCC |  |  |
| 2 | HS550 | CTAGGCATGATCTAACCCTCGG | 62 | 1641 |
|  | JM27-D | ATCCTATAGTCGAGCCCAAGC |  |  |
| 3 | S2-R | CAAGCTCTGCAGCGAGTGT | 62 | 4245 |
|  | strB Fw | GCCTGTTTTTCCTGCTCATT |  |  |
| PCR Cartography and Sanger sequencing | L1 | GGCATCCAAGCAGCAAGC | 60 | Variable |
|  | JLD2 | AAGGTATTGAGGTGATGCG |  |  |
|  | IS26 Fw | GGCATCAGTTACCGTGAGC | 56 | 338 |
|  | IS26 Rv | CGTGTTGATGAATCGTGG |  |  |
|  | HS915 | CGTGCCGTGATCGAAATCCAG | 65 | 371 |
|  | HS916 | TTCGTGCCTTCATCCGTTTCC |  |  |
|  | strA Fw | TACCGGACGAGGACAAGAGT | 58 | 165 |
|  | strA Rv | GACCCGTGCATTGAAGAGTT |  |  |
|  | strB Fw | GCCTGTTTTTCCTGCTCATT | 58 | 445 |
|  | strB Rv | CGCGTGGACGTAGTCAGTT |  |  |
|  | S2-F | TTTTCGGCATCGTCAACATA | 60 | 690 |
|  | S2-R | CAAGCTCTGCAGCGAGTGT |  |  |
|  | HS458 | GTTTGATGTTATGGAGCAGCAACG | 60 | Variable |
|  | HS459 | GCAAAAAGGCAGCAATTATGAGCC |  |  |
| Fosmid sequencing | pCC2fos Fw | GTACAACGACACCTAGAC | 50 |  |
|  | pCC2fos Rv | CAGGAAACAGCCTAGGAA | 58 |  |
