# Supplementary material for: Multidrug Resistant Uropathogenic Escherichia coli ST405 With a Novel, Composite IS26 Transposon in a Unique Chromosomal Location
Source: Front Microbiol. 2019 Jan 8;9:3212. doi: 10.3389/fmicb.2018.03212 (PMC6331395; doi:10.3389/fmicb.2018.03212)
Supplement: TABLE S4 — Relative abundance of resistance genes in ST405. [file Table_4.docx]

**File S4: Table.** Relative abundance of resistance genes in other ST405 genomes

| **Genome** | ***intI1***(identity/truncation site) | ***dfrA17*** | ***aadA5*** | ***sul1*** | ***mphA*** |  |  |
| --- | --- | --- | --- | --- | --- | --- | --- |
| 5b4d3680_7fd4_11e6_b8fe_3c4a9275d6c8 | 99/682 | - | - | - | - |  |  |
| 65fc80e0_7fd4_11e6_b8fe_3c4a9275d6c8 | - | - | - | - | - |  |  |
| 7088d9f0_7fd4_11e6_b8fe_3c4a9275d6c8 | multiple fragments | 100 | - | - | - |  |  |
| 982_ECOL |  |  |  |  |  |  |  |
| blood_09_0464* | 99/548 | 100 | 100 | 100 | 100 |  |  |
| blood_90543* | 99/548 | 100 | 100 | 100 | 100 |  |  |
| blood_90544* | 99/548 | 100 | 100 | 100 | 100 | | |
| blood_9_0292* | 99/548 | 100 | 100 | 100 | 100 |  |  |
| DHQP1301299 |  |  |  |  |  |  |  |
| Eco216 |  |  |  |  |  |  |  |
| eo1776 | 99/682 | 100 | 100 | 100 | - |  |  |
| EuSCAPE_ES009 |  |  |  |  |  |  |  |
| EuSCAPE_HU034 | 99 | 100 | 100 | 100 | 100 |  |  |
| EuSCAPE_NO011 | 99 | 100 | 100 | 100 | 100 |  |  |
| GN02215 | 99/798 | 100 | 100/158 | - | - |  |  |
| GN04682 |  |  |  |  |  |  |  |
| HICF646 | 99 | 100 | 100 | 100 | 100 |  |  |
| IHD232_15* | 99/746 | 100 | 99 | 100 | 100 |  |  |
| IR5 | multiple fragments | 100 | 100 | 100/65 | 100/89 |  |  |
| JSWP013* | 99/746 | 100 | 100 | 100 | 100 |  |  |
| PH108 | - | - | - | - | 99 |  |  |
| SAMPLE_WTCHG_320308_259190* | 99/746 | 100 | 100 | 100 | 100 |  |  |
| SCK08_36 | 99 | 100 | 100 | 100 | - |  |  |
| UPEC_1 | 99 | 100 | 100 | 100 | 100 | |  |

*Genomes* *with* *an asterisk have all genes on the same scaffold of the genome sequence.*
